# Endoscopic sleeve gastroplasty versus intragastric balloon insertion as bridge-to-surgery procedures for patients with BMI ≥50 kg/m^2^ or contraindications to primary metabolic bariatric surgery

**DOI:** 10.1177/17562848261489119

**Published:** 2026-09-08

**Authors:** Sandra Nagl, Susanne Wasserberg, Bernd Geißler, Anna Muzalyova, Helmut Messmann, Cora Aubele, Stefan Karl Goelder, Alanna Ebigbo

**Affiliations:** 1 Department of Gastroenterology, 39694 University Hospital Augsburg, Augsburg, Germany; 2 Department of General, Visceral and Transplant Surgery, 39694 University Hospital Augsburg, Augsburg, Germany; 3 Institute for Digital Medicine, 39694 University Hospital Augsburg, Augsburg, Germany; 4 39481 Ostalb-Klinikum Aalen, Aalen, Germany; 5 Department of Internal Medicine and Gastroenterology, 91789 St. Josef University Hospital Bochum, Bochum, Germany

**Keywords:** balloon, endoscopic sleeve gastroplasty, bridge-to-surgery, BMI ≥50 kg/m^2^

## Abstract

**Background:**

Metabolic bariatric surgery is the most effective obesity treatment, but high body mass index (BMI) and associated medical problems often pose surgical risks.

**Objectives:**

This study compares the safety, feasibility, and efficacy of endoscopic sleeve gastroplasty (ESG) versus intragastric balloon (IGB) as bridge-to-surgery (BTS) procedures in patients with BMI ≥50 kg/m^2^ and/or contraindications to primary metabolic bariatric surgery.

**Design:**

A retrospective analysis (2017 – 2024) included patients with BMI ≥50 kg/m^2^ undergoing ESG or IGB as a BTS procedure in a two-step approach at two German centers.

**Methods:**

The primary outcome was total body weight loss (TBWL, %) at 6 months. Secondary outcomes included absolute weight loss, BMI reduction, excess body weight loss (EBWL, %), and adverse events between the two BTS procedures.

**Results:**

Fourteen patients underwent ESG, 15 underwent IGB insertion as an intended BTS procedure. At 6 months, median %TBWL and %EBWL were significantly higher in the ESG group (17.8% (IQR, 14.5 – 22.1) vs. 8.99% (IQR, 4.6 – 14.0), p = 0.018; and 28.0% (IQR, 22.8 – 40.0) vs. 16.7% (IQR, 10.8 – 22.4), p = 0.035, respectively). IGB insertion was performed significantly faster (25 (IQR, 20.0 – 30.0) minutes (min) vs. 76 (IQR, 63.2 – 80.8) min; p < 0.01) but was associated with more mild-to-moderate adverse events (60.0% vs. 0%; p < 0.01).

**Conclusion:**

In this retrospective cohort, ESG was associated with greater short-term weight loss and fewer mild-to-moderate adverse events than IGB as a BTS procedure for patients with BMI ≥50 kg/m^2^ and/or contraindications to primary metabolic bariatric surgery (MBS).

## 1. Introduction

Obesity is a chronic disease associated with numerous associated secondary medical problems, including type 2 diabetes mellitus, dyslipidemia, hypertension, metabolic associated fatty liver disease (MAFLD), cardiovascular disease, obstructive sleep apnea, and significant morbidity and mortality.^1,2^ The global rise in obesity prevalence imposes substantial healthcare costs and socioeconomic burdens, necessitating a multifaceted approach to its prevention and management.^3^ Conservative treatment modalities, such as lifestyle modification and/or pharmacotherapy can result in modest weight loss but often fail to achieve sustained weight loss.^4^ Bariatric surgery remains the most effective and durable intervention for obesity management, offering substantial weight loss and a marked reduction in obesity-associated medical problems. Among the available surgical options, laparoscopic sleeve gastrectomy (LSG) is the most commonly performed, providing substantial weight loss with fewer adverse events.^5,6^ Despite its proven efficacy, only a small fraction of the obese population undergoes bariatric surgery due to limited access, high healthcare costs, lack of insurance coverage, and stringent eligibility criteria. Additionally, concerns about operative risks, permanent resection of gastrointestinal structures, and potential short- and long-term complications further limit its uptake.^5,7^ Over the past decades, minimally invasive endoscopic bariatric therapies (EBT) have emerged as alternative treatment modalities, bridging the gap between lifestyle interventions, pharmacological therapy, and surgery. Among EBTs, intragastric balloon (IGB) therapy, which reduces stomach volume via the placement of an inflatable balloon, is the most widely used globally to induce weight loss. Numerous studies have demonstrated the efficacy of IGBs, achieving a total body weight loss (TBWL) of approximately 10–15% at 6 months.^8–10^

Endoscopic sleeve gastroplasty (ESG) has emerged as a minimally invasive EBT gaining popularity in the treatment of obesity. Unlike IGB, ESG reduces gastric volume by creating a restrictive sleeve through endoscopic full-thickness sutures, approximating the anterior and posterior gastric walls along the greater curvature. This technique mimics the anatomical changes induced by LSG. Since its first description in 2013,^11^ multiple international multicenter studies have proven the efficacy of ESG, demonstrating a satisfactory TBWL of 15 – 20% over 1-5 years, with low adverse events rates (0.0 - 2.59%).^12–15^ Comparative studies have demonstrated the superiority of ESG over IGB, achieving significantly greater TBWL (21.3% vs. 13.9%) at 12 months.^8^ In contrast to LSG, ESG preserves the stomach’s innervation, blood supply, and hormonal function, offering the potential for reversibility and repeatability. Although ESG achieves slightly lower TBWL compared to LSG, it is associated with a lower overall incidence of adverse events and a reduced risk of new-onset gastroesophageal reflux disease (GERD).^16,17^ The safety and efficacy of minimal invasive EBTs in weight loss have primarily been demonstrated in patients with class I and II obesity, but not routinely for patients with BMI ≥50 kg/m^2^. For those patients or patients with severe associated medical problems, primary bariatric surgery may be challenging or impracticable due to factors such as significant surgical adhesions or the presence of complex abdominal hernias, which heighten the risk of postoperative morbidity and mortality.^18,19^

In this study, we evaluated the safety, feasibility, and efficacy of ESG compared with IGB as bridge-to-surgery (BTS) procedures in patients with BMI ≥50 kg/m^2^ or contraindications to primary metabolic bariatric surgery. By focusing on this particularly high-risk population, we aimed to extend the currently available evidence on endoscopic bridging strategies.

## 2. Methods

The reporting of this study conforms to the Strengthening the Reporting of Observational Studies in Epidemiology (STROBE) statement.^20^

### 2.1. Study design and patient selection

This study is a retrospective analysis of a prospectively collected database involving patients with BMI ≥50 kg/m^2^ or contraindications to primary metabolic bariatric surgery (MBS) who underwent ESG or IGB as a BTS procedure at two German hospitals (University Hospital Augsburg and Ostalb-Klinikum Aalen) between November 2017 and December 2024. The study protocol was approved by the medical ethics committee of the University of Regensburg on January 22^nd^, 2025 (Reg. Nr. 25-4058-104). The study adhered to the principles of the Declaration of Helsinki and complied with Good Clinical Practice recommendations. All authors had full access to the study data and approved the final manuscript.

We included adult patients (≥18 years) classified as BMI ≥50 kg/m^2^ who had failed prior dietary and lifestyle interventions and were deemed ineligible for primary MBS due to their BMI, severe associated medical problems, and/or an impenetrable abdominal wall. Severe obesity-associated medical problems were defined as respiratory failure, obstructive sleep apnea syndrome and/or heart failure. Complex abdominal conditions were defined as giant incisional hernias or an impenetrable abdominal wall that substantially increased operative risk as determined by the multidisciplinary obesity board.

Eligible patients underwent ESG or IGB as part of a two-step approach, involving an initial endoscopic procedure followed by subsequent surgery.

Before undergoing ESG or IGB, all patients were evaluated by a multidisciplinary team comprising a gastroenterologist, surgeon, endocrinologist, diabetologist, nutritionist, and psychologist. The decision regarding the choice of BTS procedure was made collaboratively by the patient, the treating physician, and the institutional obesity board after comprehensive education and discussion of the risks, benefits, and technical aspects of each procedure. Treatment allocation was not randomized and no predefined algorithm was applied. Instead, the decision was based on a shared decision-making process incorporating patient preference, anatomical and clinical characteristics, technical feasibility, endoscopist recommendation, and device availability at the respective institution.

### 2.2. Endoscopic procedure, peri- and postprocedural care

All patients received standardized pre-, peri-, and postprocedural care. Patients were instructed to remain nil per os (NPO) after midnight on the day of the procedure. Preoperative medications included pantoprazole 40mg and aprepitant 125mg, administered 2–3 hours prior to the procedure.

All procedures were conducted by three experienced endoscopists proficient in IGB placement and endoscopic suturing techniques. ESG procedures were performed under general anesthesia, while IGB placements were conducted either under general anesthesia or deep sedation. Carbon dioxide (CO_2_) insufflation and a standard gastroscope (GIF-H190, GIF-HQ190, GIF-EZ1500; Olympus, PA) were used, with patients positioned in either the left-lateral or supine position.

#### 2.2.1. Intragastric balloon insertion (IGB)

IGBs were performed using the Orbera balloon (formerly marketed by Apollo Endosurgery, Austin, TX; now marketed by Boston Scientific, Marlborough, USA). The balloons were inserted endoscopically following the manufacturer’s recommendations and were filled with 500 – 700mL of a 0.9% sodium chloride solution dyed with toluidine blue. The balloons were removed 6 months after insertion or earlier in cases of adverse events or intolerance.

#### 2.2.2. Endoscopic sleeve gastroplasty (ESG)

All ESG procedures were performed using either the Endomina device (Endo Tools Therapeutics, Gosselies, Belgium) or the Overstitch device (formerly marketed by Apollo Endosurgery, Austin, TX; now marketed by Boston Scientific, Marlborough, USA). A single intravenous dose of ceftriaxone 2g was administered at the induction of anesthesia as prophylactic antibiotic treatment. The procedure commenced with the placement of a full-thickness suture at the anterior wall, starting at the incisura, followed by a second suture at the same level in direction to the posterior wall. Suturing continued cranially in a sequential manner towards the gastroesophageal junction, with the fundus being spared. A total of 8–12 sutures were placed per patient to create a tubular constriction of the stomach, thereby reducing its volume.

After the BTS procedure, all patients were monitored for at least 24 hours before being discharged. Antiemetics were prescribed as needed, and 40mg pantoprazole daily was continued for 4 weeks. Patients were instructed to follow a full-fluid diet for the first 2 weeks post-procedure, followed by a pureed diet for an additional 4 weeks, and subsequently transitioned to a regular diet. Patients were followed-up by a multidisciplinary team, including a nutritionist, psychologist, surgeon, and/or gastroenterologist. Anthropometric and clinical measurements, along with a comprehensive examination, were performed at baseline prior to the EBT and during scheduled follow-up visits at 3-, 6-, and 12-months post-procedure.

### 2.3. Bariatric surgery

At a minimal interval of 7 months after the initial BTS procedure, bariatric surgery, i.e. LSG or RYGB, was performed for patients deemed eligible for surgical treatment following adequate weight loss and surgical risk assessment.

Metabolic bariatric surgery was performed according to standardized institutional techniques by experienced bariatric surgeons. During laparoscopic sleeve gastrectomy, previously placed ESG sutures and anchors were readily identifiable and did not interfere with gastric mobilization or stapling. Roux-en-Y gastric bypass was performed using a standardized laparoscopic approach. No procedure-specific modifications related to prior endoscopic treatment were required.

#### 2.3.1. Post-surgical care

Finally, the abdominal cavity is irrigated, and any residual blood or fluid is suctioned. The trocars are removed, and the port sites are closed. Postoperatively, the patient is closely monitored for signs of bleeding, infection, or leaks. An enhanced recovery pathway is typically implemented, including early ambulation, pain management, and progression from clear liquids to a structured dietary regimen. The dietary regimen is followed similarly after both IGB placement and ESG.

### 2.4. Outcomes

The primary objective of bridge-to-surgery treatment was to achieve clinically meaningful preoperative weight reduction and risk optimization before potential metabolic bariatric surgery. Consequently, treatment success was defined as successful weight loss and clinical improvement irrespective of whether patients ultimately proceeded to surgery. The primary outcome therefore was percent total body weight loss (%TBWL = [(initial weight-postprocedural weight)/(initial weight)]*100), assessed 6 months after the procedures. Secondary outcome parameters included technical success, procedure time, adverse events, change in absolute weight loss (ΔWeight, kg), change in body mass index (ΔBMI, kg/m^2^) and percent excess body weight loss (%EBWL = [(initial weight-postprocedural weight)/(initial weight - ideal body weight)]*100) between the IGB and the ESG cohort 6 months post-procedure. Ideal body weight was defined as the weight corresponding to a BMI of 25 kg/m^2^. Adverse events were classified according to the standard American Society of Gastrointestinal Endoscopy (ASGE) lexicon as mild, moderate and severe.^21^

### 2.5. Statistical analysis

Only patients with complete six-month follow-up data were included in the comparative outcome analyses. Missing data were not imputed. Data management and calculation of descriptive statistics were performed using Excel (Microsoft Corporation, Redmond, USA). Inference-statistical analysis was performed in SPSS 28.0 (Statistical Package for the Social Sciences) and R (version 4.6.1) within the RStudio integrated development environment (version 2026.07.1).

Continuous variables are presented as medians with interquartile ranges (IQRs). Proportions are reported as absolute counts and percentages, with corresponding 95% confidence intervals estimated using the Clopper–Pearson exact method (*binom* package). Between-group differences in continuous variables were analyzed using the Mann–Whitney U test, accompanied by Hodges–Lehmann estimates of the median difference and corresponding 95% confidence intervals (CI). The Mann–Whitney U test and Hodges–Lehmann estimates with 95% CI were calculated using the *stats* package, while descriptive statistics were generated using the *dplyr* package. All statistical tests were two-sided, and a p value < 0.05 was considered statistically significant.

No a priori sample size calculation was undertaken. BMI ≥50 kg/m^2^ is an uncommon condition, resulting in a limited pool of eligible participants. Therefore, all consecutive eligible patients treated during the predefined study period were included. The sample size was determined by the number of available cases rather than statistical considerations, and the findings should be interpreted as exploratory. Therefore, effect estimates and corresponding 95% CI are reported where appropriate. p-values should be interpreted cautiously and were not used as the sole basis for inference.

To assess the potential influence of baseline BMI on the primary outcome, a sensitivity analysis was performed using multiple linear regression with study group and baseline BMI as independent variables. Given the small sample size, heteroskedasticity-consistent (HC3) robust standard errors and corresponding 95% CI were calculated. Linear regression models were fitted using the *stats* package, while robust standard errors and covariance estimates were obtained using the *sandwich* package. Robust coefficient tests were performed using the *lmtest* package, and model parameters with 95% CI were reported using the *parameters* package.

## 3. Results

### 3.1. Baseline data and clinical characteristics

A total of 15 patients underwent IGB insertion, and 14 patients underwent ESG as an intended BTS-procedure. Table 1 presents the baseline characteristics of the patients in both groups. The ESG group included 6 female participants (42.9%), whereas the IGB group included 9 female participants (60.0%). The median age was 47.5 years (IQR, 40.0 – 55.8) in the ESG group and 45.0 years (IQR, 40.5 – 51.0) in the IGB group. Sex and age were comparable between both groups (p= 0.356, p= 0.747). Patients who underwent IGB placement had a similar proportion of associated medical problems (obstructive sleep apnea (64.3% vs. 73.3%, p = 0.700), diabetes mellitus type 2 (42.9% vs. 46.7%, p = 1.000), hypertension (92.9% vs. 66.7%, p = 0.169), when compared to the ESG group. All patients were considered high-risk candidates for primary surgery due to high BMI (≥50kg/m^2^) and associated medical problems such as obstructive sleep apnea syndrome, respiratory failure, heart failure, impenetrable abdomen or giant abdominal incisional hernia. The median baseline BMI was higher in the ESG group compared to the IGB group (65.8 kg/m^2^ (IQR, 58.0 – 72.4) vs. 56.5 kg/m^2^ (IQR, 54.9 – 62.3)), nearly missing significance level (p = 0.051). Both IGB and ESG procedures were technically successful in all patients (100.0% vs. 100.0%, p = 1.000). IGB insertion was performed significantly faster (25 (IQR, 20.0 – 30.0) minutes (min) vs. 76 (IQR, 63.2 – 80.8) min; p < 0.01). The median hospital stay was comparable between both groups (4 (IQR, 4.0 – 5.0) vs. 3 (IQR, 2.5 – 4.0), p = 0.063).

**Table 1.** Baseline characteristics of the study participants and the IGB and ESG procedure.

Characteristics	ESG group (n = 14)	IGB group (n = 15)	P value
Age, years Median [IQR]	47.5 [40.0 – 55.8]	45 [40.5 – 51.0]	0.747
Sex, female, n (%)	6 (42.9%)	9 (60.0%)	0.356
Baseline weight, kg Median [IQR]	208.0 [163.0 – 229.0]	178.0 [160.0 – 187.0]	0.112
Baseline BMI, kg/m^2^ Median [IQR]	65.8 [58.0 – 72.4]	56.5 [54.9 – 62.3]	0.051
Associated medical problems, n (%)			
- Obstructive sleep apnea	9 (64.3%)	11 (73.3%)	0.700
- Diabetes mellitus type II	6 (42.9%)	7 (46.7%)	1.000
- Hypertension	13 (92.9%)	10 (66.7%)	0.169
- Hyperlipidemia	8 (57.1%)	6 (40.0%)	0.466
Technical success, n (%)	14 (100.0%)	15 (100.0%)	1.00
Hospital stay, days Median [IQR]	4 [4.0 – 5.0]	3 [2.5 – 4.0]	0.0630
Procedure time, min Median [IQR]	76 [63.2 – 80.8]	25 [20.0 – 30.0]	< 0.01

### 3.2. Outcomes

Comparative outcome analyses were restricted to patients with complete six-month follow-up, resulting in 12 ESG and 13 IGB patients available for analysis (Flowchart). In the IGB cohort, two patients required early removal of the balloon (1 - 3 months after placement) due to medication-refractory intolerance. These two patients were excluded from the 6-months outcome analysis. According to the baseline characteristics of these two patients, their exclusion is unlikely to have had a meaningful impact on the study outcomes, as they were comparable to the cohort included in the analysis. The sex distribution, median age (45 years), and median procedure time (25 min) remained unchanged. The median baseline weight increased slightly from 178 to 185 kg, while the median length of hospital stay increased from 3 to 4 days. As expected, inclusion of these patients would have increased the number of moderate adverse events by two.

In the remaining 13 patients in the IGB cohort, the balloons were removed 6 months after the placement. At the 6-month follow-up, the median ΔWeight, ΔBMI, %TBWL and %EBWL from baseline were significantly higher in the ESG group compared to the IGB group (34.5 (IQR, 27.0 – 40.0) kg vs. 16 (IQR, 8.0 – 29.0) kg, p < 0.01; 11.6 (IQR, 8.39 – 12.5) kg/m^2^ vs. 4.43 (IQR, 1.96 – 9.1) kg/m^2^, p < 0.01; 17.8 (IQR, 14.5 – 22.1) % vs. 8.99 (IQR, 4.6 – 14.0) %, p = 0.018; 28.0 (IQR, 22.8 – 40.0) % vs. 16.7 (IQR, 10.8 – 22.4), p = 0.035, respectively), (Table 2).

**Table 2.** Comparison of endoscopic sleeve gastroplasty and intragastric balloon weight loss outcomes 6 months post-procedural.

Parameter	ESG group (n = 12)	IGB group (n = 13)	Hodges–Lehmann-difference (95%-CI)	P value
Change in BMI, kg/m^2^ Median [IQR]	11.6 [8.39 – 12.5]	4.43 [1.96 – 9.1]	5.52 (CI: 2.18 – 8.69)	< 0.01
Absolute weight loss, kg Median [IQR]	34.5 [27.0 – 40.0]	16 [8.0 – 29.0]	16.05 (CI: 5.00 – 25.00)	< 0.01
TBWL, % Median [IQR]	17.8 [14.5 – 22.1]	8.99 [4.6 – 14.0]	8.38 (CI: 1.63 – 13.58)	0.018
EBWL, % Median [IQR]	28.0 [22.8 – 40.0]	16.7 [10.8 – 22.4]	11.75 (CI: 1.05 – 23.05)	0.035

To address the potential confounding effect of baseline BMI being almost significant different in both groups, a sensitivity analysis using linear regression adjusted for baseline BMI was performed. After adjustment, the association between study group and %TBWL became more pronounced (adjusted β = -10.08, (CI: -15.09 to -5.07), p < 0.01) compared with the unadjusted model (β = -6.97, CI (-13.06 to -0.89), p = 0.027). Baseline BMI was independently associated with %TBWL (β = -0.43, (CI: -0.70 to -0.16); p = 0.003) (Table 3).

**Table 3.** Sensitivity analysis assessing the confounding effect of baseline BMI.

Model	Predictor	β coefficient	95% CI	P value
Unadjusted model	Group	-6.97	-13.06 to -0.89	0.027
BMI-adjusted model	Group	-10.08	-15.09 to -5.07	< 0.01
	Baseline BMI	-0.43	-0.70 to -0.16	0.003

Four ESG procedures were performed using the Overstitch device, while 8 ESG procedures were performed with the Endomina device. Device-specific subgroup analyses within the ESG cohort showed no significant differences in 6-months TBWL (16.0 (IQR, 13.3 – 19.2) % vs. 20.2 (IQR, 18.1 – 22.5) %, p= 0.270) and EBWL (24.8 (IQR, 20.5 – 35.6) % vs. 34.1 (IQR, 28.3 – 41.2) %, p= 0.270) or procedure time (80.5 (IQR, 77.8 – 81.0) min vs. 63.5 (IQR, 62.2 – 66.8) min, p= 0.054) between the Endomina and Overstitch platforms (Table 4).

**Table 4.** Comparison of outcomes according to ESG suturing platform.

Parameter	Endomina (n = 8/12)	Overstitch (n = 4/12)	Hodges–Lehmann-difference (95%-CI)	P value
TBWL 6 months, % Median [IQR]	16.0 [13.3 – 19.2]	20.2 [18.1 – 22.5]	-3.9 (CI: -11.980 – 4.790)	0.270
EBWL 6 months, % Median [IQR]	24.8 [20.5 – 35.6]	34.1 [28.3 – 41.2]	-6.5 (CI: -24.270 – 12.250)	0.270
Procedure time, min Median [IQR]	80.5 [77.8 – 81.0]	63.5 [62.2 – 66.8]	16.3 (CI: 5.000 – 21.000)	0.054

In total, 8 (53.3%) of the IGB cohort and 4 (28.6%) of the ESG cohort proceeded to surgery (LSG or RYGB) as a second step 7 – 11 months after the initial BTS procedure (Table 5). Eight patients in the ESG cohort declined sequential surgery due to sufficient primary weight loss. The remaining two ESG patients were lost to further follow-up after the 6-months visit. Additionally, five patients in the IGB cohort were lost to further follow-up after the 6-month visit, and two patients refused sequential surgery due to adequate primary weight loss at that time.

**Table 5.** Metabolic bariatric surgery.

Parameter	ESG group (n = 14)	IGB group (n = 15)	P value
No surgery, n (%) CI **(Clopper–Pearson)**	10 (71.4%) CI (0.42 – 0.92)	7 (46.7%) CI (0.21 – 0.73)	0.139
• Declined surgery, n (%) CI **(Clopper–Pearson)**	8 (57.1%) CI (0.289 – 0.823)	2 (13.3%) CI (0.017 – 0.405)	0.021
• Loss to follow-up, n (%) CI **(Clopper–Pearson)**	2 (14.3%) CI (0.018 – 0.428)	5 (33.3%) CI (0.118 – 0.616)	0.390
MBS, n (%)	4 (28.6%) CI (0.08 – 0.58)	8 (53.3%) CI (0.27 – 0.79)	0.264
• LSG, n (%) CI **(Clopper–Pearson)**	4 (28.6%) CI (0.08 - 0.58)	4 (26.7%) CI (0.08 – 0.55)	1.000
• RYGB, n (%) CI **(Clopper–Pearson)**	0 (0.0%) CI (0.00 – 0.23)	4 (26.7%) CI (0.08 – 0.55)	0.100

Among patients proceeding to definitive metabolic bariatric surgery, the median operative time for LSG was 108 (IQR, 102.8 – 110.0) min and 124 (IQR, 116.0 – 136.0) minutes for RYGB. The median postoperative hospital stay was 7 days. Median TBWL after definitive surgery was 21.5 (IQR, 20.5 – 23.5) and 25 (IQR, 23.5 – 27.2) after LSG at 6 and 12 months, respectively, and 27 (IQR, 26.5 – 27.5) and 32.0 (IQR, 30.5 – 32.5) after RYGB at 6 and 12 months, respectively. Notably, all patients who underwent RYGB had received IGB as the bridging procedure, whereas no RYGB procedures were performed in the ESG group. Therefore, comparisons between LSG and RYGB should be interpreted with caution, as the effects of the definitive surgical procedure and the preceding bridging strategy cannot be clearly separated. Median TBWL after definitive surgery according to BTS procedure was 22.0 (IQR, 20.5 – 24.2) and 26.0 (IQR, 24.2 – 28.0) for the ESG group at 6 and 12 months, respectively, and 25 (IQR, 21.8 – 26.2) and 26.5 (IQR, 23.5 – 29.8) for the IGB group at 6 and 12 months, respectively (Tables 6 and 7).

**Table 6.** Post-metabolic bariatric surgery outcomes by surgery type.

Parameter	RYGB (n = 4)	LSG (n = 8)	Hodges–Lehmann-difference (95%-CI)	P value
TBWL 6 months after surgery, % Median [IQR]	27 [26.5 – 27.5]	21.5 [20.5 – 23.5]	-5.0 (CI: -9.00 – 1.00)	0.064
TBWL 12 months after surgery, % Median [IQR]	32 [30.5 – 32.5]	25 [23.5 – 27.2]	-6.3 (CI: -11.00 – -1.00)	0.031
Procedure time, min Median [IQR]	124.0 [116.0 – 136.0]	108.0 [102.8 – 110.0]	-18.38 (CI: -51.00 – 4.00)	0.126
Hospital stay, days Median [IQR]	7.0 [6.5 – 7.5]	7.0 [6.0 – 7.0]	0.0 (CI: -2.00 – 1.00)	0.664

**Table 7.** Post-metabolic bariatric surgery outcomes by BTS procedure (ESG vs IGB).

Parameter	ESG group (n = 4)	IGB group (n = 8)	Hodges–Lehmann-difference (95%-CI)	P value
TBWL 6 months after surgery, % Median [IQR]	22.0 [20.5 – 24.2]	25 [21.8 – 26.2]	-2.0 (CI: -7.00 – 5.00)	0.670
TBWL 12 months after surgery, % Median [IQR]	26.0 [24.2 – 28.0]	26.5 [23.5 – 29.8]	-0.5 (CI: -7.00 – 6.00)	0.932
Procedure time, min Median [IQR]	110.0 [108.0 – 114.0]	108 [98.0 – 114.0]	4.71 (CI: -28.00 – 30.00)	0.551
Hospital stay, days Median [IQR]	7 [6.75 – 7.0]	7 [6.0 – 7.25]	0.0 (CI: -1.00 – 1.00)	1.00

### 3.3. Adverse events

Mild-to-moderate adverse events, such as nausea, vomiting, or abdominal pain were common after IGB placement and were controlled with anti-emetic medication and/or analgesic medication. These events differed significantly between the two groups (60.0% vs. 0%, p < 0.01). No severe adverse events requiring invasive intervention were observed in either BTS group. Two patients in the IGB cohort required early removal of the balloon due to refractory intolerance (Table 8).

**Table 8.** Adverse events.

Parameter	ESG group (n = 14)	IGB group (n = 15)	P value
Adverse Events, n (%)	0 (0.0%) CI (0.000 – 0.231)	9 (60.0%) CI (0.323 – 0.837)	< 0.01
• Severe Adverse events, n (%)	0 (0.0%) CI (0.000 – 0.231)	0 (0.0%) CI (0.000 – 0.218)	1.000
• Mild-to-moderate Adverse Events, n (%)	0 (0.0%) CI (0.000 – 0.231)	9 (60.0%) CI (0.323 – 0.837)	< 0.01

**Figure 1.**
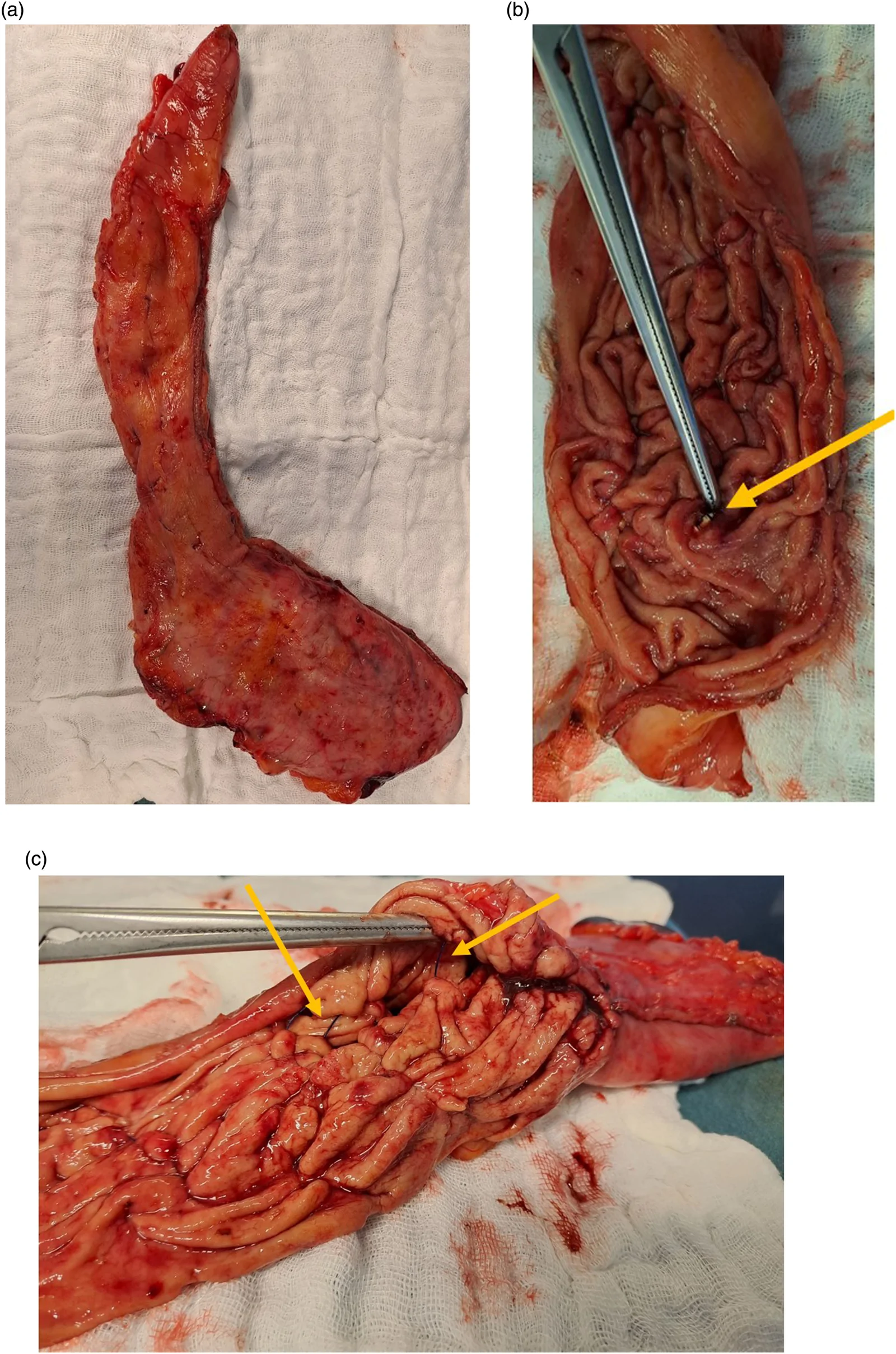
(A) – (C): Laparoscopic sleeve gastrectomy specimen with identification of endoscopic sleeve gastrectomy sutures and anchors (marked with arrows).

No intraoperative complications were observed during the surgical procedures in either study group (0 patients (0.0%) in the ESG group and 0 patients (0.0%) in the IGB group, p= 1.000). In cases where prior ESG had been performed, sutures and anchors were successfully identified intraoperatively within the abdominal cavity, obviating the need for intraoperative esophagogastroduodenoscopy (EGD) to prevent leakage or stapler misfires at the resection site (Figure 1). Similarly, 30-day postoperative morbidity and mortality rates were 0 patients (0.0%) and 0 patients (0.0%) in the respective groups (p= 1.000). Thus, no between-group differences in perioperative safety outcomes could be identified.

## 4. Discussion

Obesity is a chronic disease requiring effective long-term treatment. While MBS remains the most effective intervention, limited accessibility and concerns regarding surgical morbidity have increased interest in EBTs such as IGB placement and ESG, both of which have demonstrated favorable efficacy and safety profiles.^5,8,22–29^

IGB is a well-established EBT associated with approximately 10–12% TBWL at 12 months, including in patients with BMI ≥50 kg/m^2^ or contraindications to primary MBS.^30,31^ Since its introduction by Abu Dayyeh et al., ESG has emerged as an effective alternative, with prospective and multicenter studies demonstrating 15–21% TBWL and sustained efficacy for up to five years.^11,12,14,15,32–34^ Consequently, current ASGE/European Society of Gastrointestinal Endoscopy (ESGE) guidelines endorse endoscopic bariatric and metabolic therapies for adults with a BMI ≥27 kg/m^2^.^35^ Previous comparative studies consistently reported greater weight loss after ESG than after IGB (17.5–21.3% for ESG vs 10.4–13.9% for IGB at 12 months).^8,29^

Our findings extend these observations to a highly selected cohort of patients with BMI ≥50 kg/m^2^ or contraindications to primary MBS. For those patients, MBS such as RYGB and LSG are associated with increased technical difficulty, higher risk of intraoperative complications and conversions, longer operative times, increased postoperative morbidity and mortality and extended postoperative hospital stay.^18,19,36,37^ These challenges are primarily attributed to the presence of multiple and severe associated medical problems, increased thickness of the abdominal wall, omentum and mesenteries, as well as hypertrophy of the left hepatic lobe, which can increase hepatic fragility.^38,39^ In a study based on a national database from the United States, the rates for conversion, complication, and mortality were statistically significantly higher for patients with BMI ≥50kg/m^2^.^19^ Another study on high-risk and high-BMI patients undergoing LSG showed that 15% of the cases experienced short-term complications, such as pulmonary embolism, gastric leak, and bleeding.^40^ Compared with ESG, LSG provides greater weight loss (23.6% vs. 17.1% TBWL) but at the expense of higher overall complication rates (16.9% vs. 5.2%) and a markedly increased risk of de novo GERD (14.5% vs. 1.9%).^16^

To our knowledge, this is the first full-length comparative study specifically evaluating ESG and IGB as BTS procedures in patients with BMI ≥50 kg/m^2^ or contraindications to primary metabolic bariatric surgery. In our study, both ESG and IGB were technically successful as a BTS procedure in all patients. The 6-month mean %TBWL was 10.86 ± 7.74 for the IGB cohort and 17.83 ± 5.73 for the ESG cohort, respectively, aligning with previous literature.^8,10,12,14,29,31,33,41^

ESG resulted in significantly greater %TBWL than IGB, consistent with previous comparative studies and meta-analyses. A recent meta-analysis by Huang et al. reported an increasing relative benefit of ESG with higher baseline BMI in an exploratory study-level meta-regression.^42^ However, as this analysis was based on study-level rather than individual patient data, the authors acknowledged the potential for ecological fallacy, and these findings should not be interpreted as direct evidence of a patient-level association. Previous studies have suggested that baseline BMI may be associated with greater weight loss following endoscopic bariatric therapies.^12,42^ Because baseline BMI differed between the two groups, we performed a sensitivity analysis adjusting for baseline BMI. The association between study group and %TBWL remained significant and became more pronounced after adjustment, suggesting that the observed group difference is unlikely to be explained by baseline BMI alone. Nevertheless, given the observational study design and limited sample size, residual confounding by unmeasured factors cannot be completely excluded.

In the IGB cohort, all balloons were routinely removed six months after placement, except for two patients who required early removal because of intolerance. We intentionally restricted the comparative analysis to the six-month treatment period, as this represents the standard duration of balloon therapy. However, weight regain following balloon removal is a well-recognized limitation of IGB treatment, with previous studies reporting partial or complete weight regain in a substantial proportion of patients within the first months after explantation.^43,44^ Consequently, the timing of definitive MBS following balloon removal is likely to be clinically relevant and may influence long-term outcomes. Unfortunately, long-term post-removal weight trajectories were not systematically available in our retrospective cohort and should be investigated in future prospective bridge-to-surgery studies. Despite the anticipated weight regain, IGB may still serve as a viable BTS procedure due to its ease of placement and rapid implementation. However, ESG, being a single-stage procedure, resulted in greater and more sustained weight loss, may provide a more durable BTS strategy. Additionally, ESG has been shown to induce physiological changes in gastric function and appetite regulation, potentially addressing compensatory counter-regulatory mechanisms that contribute to weight recidivism.^45^ Interestingly, a higher proportion of patients in the ESG cohort (57.1%) compared to the IGB cohort (13.3%) declined second-step MBS because they considered the achieved weight loss sufficient (P = 0.021). This observation highlights that successful BTS treatment should not exclusively be defined by progression to surgery but may also include clinically meaningful weight reduction that alters the patient’s therapeutic decision-making.

ESG is technically more complex than IGB placement and requires a longer learning curve. One study showed that approximately 35 procedures by a single operator are needed to achieve efficiency in terms of reducing procedure time, while mastery - defined as the absence of outliers - was attained after 55 procedures.^46,47^ Although our study included patients at the beginning of the ESG learning curve, which contributed to a longer procedure time, the comparable TBWL rates at 6 months align with existing literature. The longer procedure time associated with ESG may also have clinical implications beyond procedural efficiency. Patients with BMI ≥50 kg/m^2^ frequently present with obstructive sleep apnea, obesity hypoventilation syndrome, and cardiovascular associated medical problems, potentially increasing the risk of prolonged anesthesia. Although no anesthesia-related complications occurred in our cohort, this aspect should be considered during patient selection and procedural planning. Nevertheless, the longer initial procedure time may be offset by the fact that ESG represents a single intervention, whereas IGB requires both implantation and scheduled removal under endoscopic guidance. The comparable outcomes observed for the Overstitch and Endomina platforms indicate that both devices provide similar short-term efficacy and procedural performance within ESG, suggesting that factors other than the suturing platform itself may have a greater influence on treatment outcomes.

From a health-economic perspective, ESG requires dedicated suturing devices, specialized operator training, and longer procedure times compared with IGB placement. In contrast, IGB insertion is technically simpler and more widely available but requires a second endoscopic intervention for balloon removal and was associated with a higher rate of treatment-related intolerance in the present cohort. Although formal cost-effectiveness analyses were beyond the scope of this study, the greater durability of weight loss and single-stage nature of ESG may partially compensate for its higher initial procedural costs. Future prospective studies should incorporate economic analyses when comparing bridge-to-surgery strategies.

ESG was associated with fewer mild-to-moderate treatment-related adverse events than IGB, whereas no severe adverse events occurred in either group. Mild-to-moderate adverse events, such as abdominal pain, nausea, and vomiting, were observed in the IGB cohort. Most of these were managed conservatively with medications. Two patients required early removal of the IGB due to intolerance. Literature reports adverse events following IGB placement in up to 28.2% of cases, with a serious adverse event rate of 10.5%.^48^ In comparison, ESG has a significantly lower adverse event rate, as reported by Fayad et al. (17% for IGB vs. 5.2% for ESG, P = 0.048).^8^ The incidence of moderate or severe adverse events following ESG ranges from 0.0% to 2.7%, with the most common being upper gastrointestinal hemorrhage and perigastric fluid collection.^12–14,32^ In our cohort, no adverse events were noted following ESG placement. Although the IGB cohort experienced a higher rate of mild-to-moderate adverse events, such as retching, nausea, and vomiting, the length of hospital stay was not affected. However, the higher incidence of these adverse effects in the IGB group emphasizes the importance of proper pre-procedure evaluation and ongoing vigilance for potential complications.

There is limited evidence regarding the safety and feasibility of MBS following ESG. In particular, LSG may be technically challenging because of the presence of sutures, anchors, and cinches along the greater curvature and the potential development of fibrosis.^49,50^ In our cohort, however, definitive MBS after both ESG and IGB was technically feasible and associated with excellent short-term perioperative outcomes. All LSG procedures following ESG were completed without intraoperative complications, with sutures readily identifiable from the abdominal cavity and without the need for intraoperative esophagogastroduodenoscopy. No intraoperative complications or 30-day morbidity or mortality occurred, and postoperative weight loss after LSG and Roux-en-Y gastric bypass was consistent with published benchmarks. Although stapler misfire remains a potential concern in patients with prior ESG and intraoperative esophagogastroduodenoscopy may facilitate identification of anchors and sutures, our findings support the feasibility and short-term safety of endoscopic BTS strategies. Nevertheless, these results should be interpreted cautiously given the limited number of patients undergoing definitive surgery.

Overall, for patients with BMI ≥50 kg/m^2^ or contraindications to primary MBS, ESG may be a more attractive option due to its ability to produce more significant weight loss without increasing surgical risk.

The rapidly evolving field of anti-obesity pharmacotherapy has substantially changed the therapeutic landscape. Highly effective GLP-1 receptor agonists and dual GIP/GLP-1 agonists, including semaglutide and tirzepatide, have demonstrated weight-loss outcomes approaching those of endoscopic bariatric therapies and may represent alternative bridging strategies before metabolic bariatric surgery in selected patients. However, long-term adherence, treatment costs, gastrointestinal adverse events, and the potential for weight regain after discontinuation remain important limitations.^51^ Endoscopic BTS procedures provide a durable anatomical intervention without requiring continuous medication and may therefore remain particularly attractive in patients with BMI ≥50 kg/m^2^ or contraindications to primary metabolic bariatric surgery.

A key strength of our study is that, despite its retrospective design, both groups followed a standardized care program before and after the procedure, and all procedures were performed by only three endoscopists. Regular follow-up appointments, along with nutritional and psychological support, are crucial for successful weight loss and essential for adequately comparing the two procedures.

However, there are some limitations that must be addressed. The relatively small sample size and prolonged inclusion period reflect the highly selected nature of this patient population and inevitably limit statistical power, increasing the risk of both type I and type II errors. Owing to the exploratory design and limited sample size, the findings should be interpreted as hypothesis-generating rather than confirmatory. Larger multicenter studies are required to validate these observations. Loss to follow-up further limits the interpretation of secondary surgical outcomes and may have introduced attrition bias. Therefore, long-term outcomes should be interpreted cautiously. Patients were included at the beginning of the ESG learning curve. However, it is unlikely that this impacted the primary outcomes, as several studies have demonstrated that ESG has a relatively short learning curve, and the weight loss results in our study were consistent with those reported in the literature. The only notable difference in our study was a longer procedure time observed in the ESG cohort compared to the procedure times reported in the literature.^34^ Furthermore, treatment allocation was based on individualized multidisciplinary decision-making rather than predefined criteria, introducing the potential for selection bias and confounding by indication. Furthermore, long-term follow-up after IGB removal was not uniformly available, precluding assessment of post-explantation weight regain and its potential influence on subsequent MBS.

## 5. Conclusion

In this exploratory retrospective cohort, ESG was associated with greater short-term weight loss and fewer mild-to-moderate adverse events than IGB, while both procedures were technically feasible as BTS strategies in patients with BMI ≥50 kg/m^2^ or contraindications to primary metabolic bariatric surgery. These findings should be confirmed in larger prospective comparative studies.

## Appendix

### Abbreviations

BMI: Body mass index
ΔBMI: Change in body mass index
ΔWeight: Absolute weight loss
ASGE: American Society of Gastrointestinal Endoscopy
BTS: Bridge-to-surgery
CI: Confidence interval
CO2: Carbon dioxide
EBT: Endoscopic bariatric therapy
EBWL: Excess body weight loss
EGD: Esophagogastroduodenoscopy
ESG: Endoscopic sleeve gastroplasty
ESGE: European Society of Gastrointestinal Endoscopy
GERD: Gastroesophageal reflux disease
HC3: Heteroskedasticity-consistent
IGB: Intragastric balloon
IQR: Interquartile range
LSG: Laparoscopic sleeve gastrectomy
MAFLD: Metabolic associated fatty liver disease
MBS: Metabolic bariatric surgery
Min: Minutes
NPO: Nil per os
RYGB: Roux-en-Y gastric bypass
SPSS: Statistical Package for Social Sciences
TBWL: Total body weight loss.
